# Operating-Condition Residual Normalization: A Bio-Inspired Operator for Sensor Integrity Monitoring in Automated Vehicles

**DOI:** 10.3390/biomimetics11090657

**Published:** 2026-09-12

**Authors:** Mehmet Bilban, Onur İnan

**Affiliations:** 1 Seydişehir Vocational School, Necmettin Erbakan University, 42360 Konya, Türkiye; mbilban@erbakan.edu.tr; 2 Department of Computer Engineering, Faculty of Technology, Selçuk University, 42130 Konya, Türkiye

**Keywords:** operating-condition residual normalization (OCRN), bio-inspired algorithms, biomimetic intelligence, constrained multi-objective design, algorithm benchmarking, machine-learning-assisted optimization, efference copy, reafference principle, minimum detectable fault, sensor integrity

## Abstract

Bio-inspired integrity monitors for automated vehicles are reported as single pooled detection figures, which conflates algorithmic performance with evaluation protocol. Transferring the reafference principle to inertial-channel integrity, we identify what actually governs the reported figure. A single-track forward model is identified from the data, and its residual is standardized by Operating-Condition Residual Normalization (OCRN), a bin-wise operator conditioned on an observable operating point; the design is resolved by an exhaustive constrained search and each component is isolated using ablation. Across 224,638 samples spanning five towns and four friction levels, the residual scale varies by a factor of 158, and OCRN recovers 21.8 F_1_ points, more than the detection rule, the encoder, and the biological attenuation gate combined. A cross-comparison confirms this: changing the detection rule moves the result by 0.09 points, while changing the normalization moves this by 15 to 19. The monitor attains 97.34% precision, 76.35% recall, and 85.57% F_1_ at a 0.76% false-alarm rate when counting every injection, and 97.30/93.99/95.62% above a 3σ detectability floor when covering 80.1% of them. The floor scales with forward-model error, which is correlated with but not determined by the road friction, and the gate, the most explicitly biological element, is not selected once the residual is conditionally normalized, with its best setting gaining at most one F_1_ point at nearly twice the false-alarm rate.

## 1. Introduction

An automated vehicle infers its own motion from inertial measurements. The yaw rate and lateral acceleration enter the electronic stability control, with dead-reckoning between satellite fixes, a prediction step of sensor fusion, and plausibility checks that guard higher-level functions. This makes the inertial channel structurally privileged: a great deal of vehicle behavior is conditioned upon it while comparatively little checks it.

The channel is also structurally exposed. Inertial measurement units are commodity components procured from tier-two suppliers, mounted in accessible locations, and connected over buses shared with dozens of other nodes. Physical signal-injection attacks against microelectromechanical inertial sensors have been demonstrated repeatedly [1], and bus-level false-data injection requires only the access that a service operator or an aftermarket device already possesses [2]. Regulations UN R155 [3] and ISO/SAE 21434 [4] both require that such interfaces be analyzed and monitored, though neither prescribes how.

The conventional answer is a model-based residual test. A vehicle model predicts the measurement, the prediction is subtracted, and a statistic of the remainder is compared against a threshold. This approach is mature, as chi-squared tests, cumulative-sum procedures, and their windowed and adaptive-threshold variants are standards [5,6], and the conditions under which an attacker may remain below the alarm threshold have been characterized precisely [7,8]. The residual-based approach is not what is missing.

What is missing, and what this paper addresses, is a systematic account of what determines the performance such a monitor actually achieves. The reported detection figures in the literature were typically single pooled numbers from a single operating condition, obtained with a threshold calibrated on the same data, and without an explicit statement of which faults were physically detectable in the first place. Under these conventions, the same detector can be made to report almost any figure.

### 1.1. The Biological Analog and Its Limits

The problem of distinguishing self-caused from externally caused sensation is old and solved. The reafference principle [9] holds that, with a copy of the motor command, the efference copy is routed to sensory centers, where an internal forward model predicts the sensory consequence of the organism’s own action [10]. Sensation matching the prediction is reafferent and is attributed to self-motion [11]; the unexplained remainder is exafferent and is attributed to the world [12]. Vertebrate vestibular processing implements this directly [13], and the mechanism includes a second element frequently overlooked in engineering transfers; efferent discharge also modulates the gain of peripheral receptors during self-generated movement, reducing sensitivity precisely when self-motion would otherwise saturate the channel [14].

When mapped onto a vehicle, the actuator commands are the efference copy, a vehicle model is the forward internal model, and the residual is the exafference. In its subtraction step, this mapping is mathematically identical to model-based residual generation, a point we state plainly rather than obscure. Its distinctive contribution is the second element: a command-dependent modulation of detector sensitivity. We implement this as a maneuver-dependent attenuation gate and, unusually for a bio-inspired study, report that the optimizer sets both of its coefficients to zero.

This result points to where the contribution of this work actually lies. The novelty of this work lies not in the detection rule but in the three methodological results that apply to any model-based integrity monitor. First, we establish residual heteroscedasticity as the dominant design factor and provide a conditional-normalization treatment for it. Second, we transfer the minimum-detectable-fault construct from fault diagnosis to attack detection, and we pair it with a coverage metric so that recall figures become interpretable. Third, we show that the detectability floor is a function of the operating condition rather than a constant, which changes what a detectability specification must contain. Each of these is measured rather than asserted, and each is accompanied by an ablation that isolates its effects.

The element of the biological principle that is reproduced computationally is specific and worth stating plainly: it is the comparison of a measured afferent signal against a prediction generated from a copy of the motor command, with the unexplained part of the difference attributed to the external world. This comparison, and not the spiking substrate or the attenuation mechanism, is the computational content of the reafference principle. The advantage it confers over a purely data-driven detector is that the prediction is grounded in an actuation command the vehicle already possesses, so no model of the attack is required and no labeled attack data are needed to calibrate the monitor. Section 7.2 reports where the analogy stops being useful.

### 1.2. Contributions

(i) The OCRN operator. We establish that the yaw-rate residual scale varies by a factor of 158 across the operating envelope, from 0.0026 rad s^−1^ above 15 m s^−1^ to 0.4121 rad s^−1^ in the 2–5 m s^−1^ transient band, and that 46.7% of the urban telemetry collected in this study is recorded at standstill. We introduce a bin-wise conditional standardization that recovers 21.8 F_1_ points more than the detection rule, the encoder, and the biological gate combined. This is the single largest contributor in the study and, to our knowledge, has not previously been applied to vehicle integrity monitoring.

(ii) A detectability floor with reported coverage. We adapt the minimum-detectable-fault construct from fault diagnosis to attack detection, derive the floor from the residual scale and the target false-alarm rate, sweep it over four levels, and report the performance both with and without it, together with the fraction of injected samples it excludes. Recall figures in the literature are routinely reported without this pairing and are therefore not comparable across studies.

(iii) A condition-dependent detectability specification. We show that the floor scales with forward-model error, which correlates with tire–road friction but is not determined by it: the coverage tracks the residual scale at r = −0.946 across all scenarios with the friction level only at r = +0.289. We therefore propose the residual scale at the operating point, which is measurable at runtime, alongside δmin(μ, v) as the appropriate object for a quantitative impact rating under ISO/SAE 21434.

(iv) An identified rather than assumed forward model. Vehicle geometry is read from the simulator physics model; only the understeer gradient and steering-actuator time constant are fitted over four candidate structures, with friction-conditioned parameters and a leave-one-town-out learned correction that raises the worst-case performance by 14.4 points.

(v) A reproducible evaluation protocol and three negative findings. Strictly causal filtering, offline nominal calibration, an exhaustive search of each baseline design space, pooled and per-scenario reporting with worst-case columns, and a paired non-parametric statistical treatment are provided. We further report three results unfavorable to the framing that motivated the work: the lateral-acceleration channel degrades the performance and receives zero weight; the maneuver-dependent attenuation gate is not selected once the residual is conditionally normalized, because the F_1_ it gains comes at the cost of false alarms; and the learned correction improves robustness rather than accuracy.

### 1.3. Scope

This is a detector characterization study conducted on recorded telemetry. Injections are applied to logged inertial signals; the vehicle operates under the simulator’s autopilot, and the injected signal does not propagate through the control loop. No claim is made regarding trajectory deviations, lane departures, or any safety outcomes. Closed-loop propagation is identified as the next study’s focus and is stated as the principal limitation in Section 7.5.

## 2. Related Work

### 2.1. Model-Based Residual Detection and Stealthy Attacks

Residual-based fault detection has a long lineage [6,15], and its adaptation to adversarial settings is well-developed. The reachable set of vehicle states an attacker can induce while maintaining an alarm rate close to nominal has been characterized for both static and dynamic detectors [7], and synthesis tools exist that can minimize this set [8]. The advantage of dynamic over static detection is therefore already quantified in the literature and is not claimed here. Likewise, maneuver-dependent thresholding for vehicle-dynamic diagnostics, which selects diagnostic thresholds according to an estimated maneuver state derived from the yaw rate, speed, and steering angle, is established in the patent record [16] and predates this work by more than fifteen years. Our contribution is not the existence of a command-conditioned gate but the measurement of what it costs, what it exposes, and what dominates it. As Section 6.5 shows, the gate is not selected at the identified operating point.

Related work has addressed vehicle motion control under delayed and noisy measurements using deep learning combined with an unscented Kalman predictor [17], but with a fixed, non-adapting controller and without explicit detectability analysis.

### 2.2. Bio-Inspired and Neuromorphic Detection in Vehicles

Spiking and brain-inspired methods have been applied both to autonomous-driving control [18,19] and to sensor fault diagnosis [20]. The membrane-learnable residual spiking network of Wang and Li [21] diagnoses faults injected into accelerator-pedal, brake-pressure, and steering-angle signals on a public autonomous-driving dataset with 96.9–99.1% classification accuracy. This work is the closest neighbor to ours and differs in three respects that define our position: it is an offline multi-class classifier rather than a causal monitor; it treats the inertial channel as the trusted auxiliary reference used to detect anomalies in actuator-command signals, which is the inverse of the problem addressed here; and it contains no forward model and, consequently, no notion of self-generated versus externally caused sensations. Hyperdimensional and recurrent-spiking anomaly detectors for automotive sensor attacks operate on reconstruction errors or on network traffic [22,23,24] and likewise contain no causal model of vehicle motion.

In prior work, we examined learning-based control and sensor-fusion estimation for autonomous vehicles from the performance side, using policy optimization and chaotic ensemble methods, respectively [25,26]; the present study addresses the complementary question of what happens when the inertial channel feeding such estimators is itself corrupted.

### 2.3. Efference Copy in Engineering Systems

Efference copy and corollary discharge have been transferred to robotics chiefly for gaze stabilization and manipulator control. We are not aware of the prior use of the reafference principle as an integrity primitive for vehicle inertial channels; however, we emphasize that the residual generation implied by the principle is standard, and that our claim is confined to the characterization study rather than to novelty of the subtraction itself. Table 1 compares the present work against the closest prior research on the dimensions that determine whether a reported detection figure is interpretable.

### 2.4. Condition-Dependent Normalization and Scheduled Thresholds

Standardizing a residual by a condition-dependent scale is not new, and the operator proposed here should be positioned against two established lines of work. In estimation, the normalized innovation squared test scales the innovation of a filter by the covariance the filter itself propagates, which makes the test statistic comparable across operating points whenever that covariance is a faithful description of the innovation. In vehicle diagnostics, maneuver- and speed-scheduled thresholds achieve a similar effect by prescribing a threshold as a function of the measured state, and such schedules have long been established in production-fault-detection functions.

What the present operator adds is not conditional normalization as such but the source of the scale. It requires neither a propagated covariance nor a scheduling law, only attack-free data that span the operating range, and it therefore applies in the regime studied here, where the residual scale is governed by forward-model error rather than by a known measurement covariance and where no covariance model of that error is available. The cost is symmetric: the operator provides no scale outside the range covered by the calibration data, and it inherits any bias present in that data. We accordingly frame the contribution of this paper as the measurement of how much conditional normalization matters in this setting, quantified in Section 6.6, rather than as the introduction of a previously unavailable mechanism.

A related construct exists on both sides of the literature and is rarely connected across them. In model-based fault diagnosis, the minimum detectable fault is a standard object, where the residual-generation literature characterizes the smallest deviation of a given residual and threshold as separate from the nominal condition [5,6], and analytical redundancy relations make the same limit explicit in structural terms [15]; the literature on incipient faults, as discussed in Section 6.3, is concerned precisely with deviations that approach that limit. The security literature has an analogous object that approaches from the attacker side: the characterization of what a given detector cannot see, expressed as the reachable set of states an adversary can induce without triggering an alarm [8], and the corresponding analysis of a specific detector statistic [7]. The two lines describe the same limit from opposite directions, yet neither is routinely reported alongside recall as a coverage figure, such that a reported recall value carries no information about how much of the injected population was detectable in the first place. Section 5.6 adopts the fault-diagnosis construct for the attack setting and reports coverage explicitly for that reason.

## 3. Problem Formulation and Threat Model

### 3.1. System

The vehicle is described by a single-track model with a wheelbase L, longitudinal speed v, and road-wheel steering angle δ. The monitor observes the actuator command, comprising steering, throttle, and brake; the measured yaw rate ω_z_ and lateral acceleration a_y_ from the inertial unit; and the vehicle speed. Sampling is synchronous at 100 Hz.

### 3.2. Threat Model

We assume an adversary capable of modifying inertial measurements in transit or at source but incapable of modifying actuator commands. We explicitly reject a cryptographic justification for this asymmetry as circular; were an authenticated bus available for actuator commands, it would equally be available for the inertial unit. The asymmetry on which we rely is architectural and supply-chain based. Actuator commands are generated by, and consumed within, the domain controller that also hosts the monitor, and need not traverse an external bus to reach it [27]. The inertial unit is, by contrast, a discrete, externally sourced, field-replaceable component on a shared bus, accessible during service operations and reachable both by aftermarket devices [2] and by physical signal injection [1]. This is a matter of degree rather than of guarantee, and the consequences of its violation are analyzed in Section 7.5.

The practical significance of this threat has been demonstrated for multi-sensor-fusion localization, where spoofing of a single positional channel produces unbounded drift in high-level autonomous driving stacks [28]. The adversary is assumed to know the vehicle model but cannot adapt to the monitor’s internal state. Adaptive adversaries that optimize against the attenuation gate are out of scope and are identified in Section 7.5 as the principal open threat.

### 3.3. Attack Classes

Three canonical injection profiles are used, spanning the persistence–frequency plane. They constitute a characterization instrument rather than a claim of adversarial completeness.

The three profiles are summarized in Table 2.

Corresponding lateral-acceleration injections of 1.2 sin(·), 1.8, and 0.25 (t − 72) m s^−2^ are applied for completeness, although Section 6.2 shows the acceleration channel is not used by the final monitor. Ground-truth labels mark the entire injection window. Class A1 crosses zero five times within its window and class A3 begins at zero; a proportion of the labeled samples therefore carry an injected magnitude below any achievable noise floor. This is addressed explicitly in Section 5.6 rather than absorbed silently into the recall figure.

## 4. Materials and Methods

**Figure 1:**
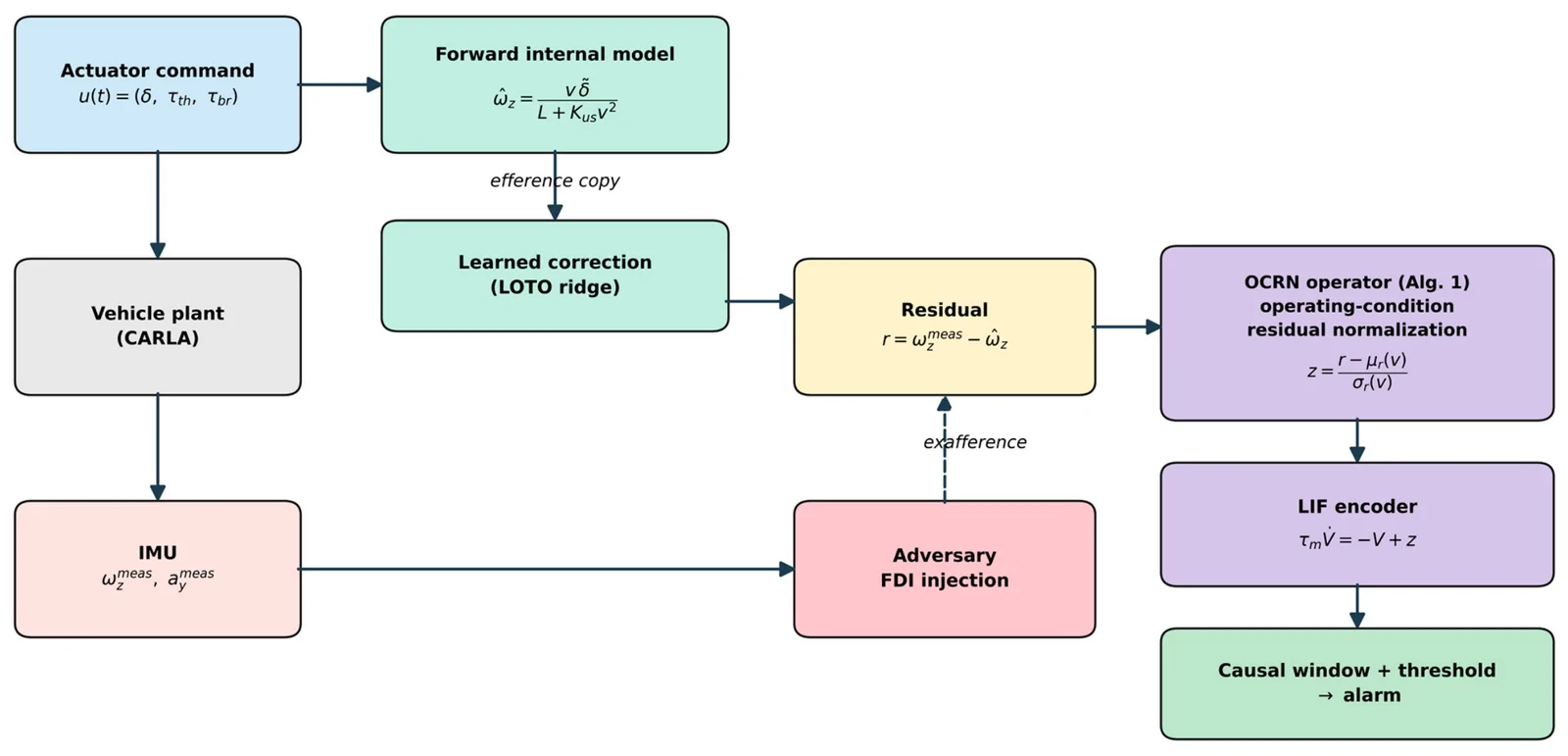
Reafference-principle monitor architecture. The actuator command serves as the efference copy driving a forward internal model; the residual between measurement and prediction constitutes the exafference. The OCRN operator (Algorithm 1) and event-driven encoding precede the threshold test.

The monitor comprises five stages: forward prediction, residual formation, OCRN, event-driven encoding, and gated accumulation with a threshold test. Each stage is causal. Figure 1 shows the architecture.

**Algorithm 1.** Operating-Condition Residual Normalization (OCRN)Calibration phase (offline, attack-free data) Input: nominal residuals r_nom [1..N], operating variable c_nom [1..N],     bin edges E [0..B], minimum bin count n_min_, variance floor s_min Output: per-bin statistics {mu_b, sigma_b}, b = 1..B 1:    for b = 1 to B do 2:     I_b <- { i:E[b-1] ≤ c_nom[i] < E[b]} 3:     if |I_b| ≥ n_min_ then 4:      mu_b <- mean(r_nom[I_b]);    sigma_b <- std(r_nom[I_b]) 5:     else 6:      mu_b <- mean(r_nom);         sigma_b <- std(r_nom) 7:     end if 8:     sigma_b <- max(sigma_b, s_min) 9:    end for
Runtime phase (online, constant time and memory per sample) Input: residual r(t), operating variable c(t), statistics {mu_b, sigma_b} Output: standardized residual z(t) 10: b    <- BIN(c(t), E) 11: z(t) <- (r(t) − mu_b)/sigma_b 12: return z(t)

The symbols used throughout this section are collated in Table 3.

### 4.1. Forward Model (Efference-Copy Pathway)

Vehicle geometry is read from the simulator physics model and is not fitted: front- and rear-wheel positions give a wheelbase of L = 3.005 m, and the front-wheel maximum steering angle gives δ_max_ = 70°. The normalized command is converted to a road-wheel angle. The selected structure is a linear single-track model, with an understeer gradient and a first-order steering-actuator lag:

(1) $$\tilde{\delta }(t)\;=\;{LPF}_{\tau s}[\delta (t)]$$

(2) $${\hat{\omega }}_{z}(t)\;=\;v(t)\;\tilde{\delta }(t)/(L+K_{us}\;{v(t)}^{2})$$

(3) $${\hat{a}}_{y}(t)=v(t){\hat{\omega }}_{z}(t)$$

Equation (2) is the steady-state yaw-rate gain of the linear bicycle model [29]; the K_us_ v^2^ term captures the speed-dependent loss of yaw gain that the purely kinematic model omits and dominates at the speeds present in the dataset. Equation (3) is the corresponding steady-state relation for lateral acceleration. Only K_us_ and τs are fitted; Section 5.2 reports the structure selection.

Equation (2) is the steady-state yaw-rate relation of the single-track model with an understeer gradient. Under the assumptions of small sideslip, linear tire behaviors, and a planar road, the lateral force balance and the yaw moment balance reduce to a first-order relation between the road-wheel angle and the yaw rate, in which the effective wheelbase is augmented by the term K_us_ v2. The first-order lag applied to the commanded angle represents the steering actuator, whose time constant is identified from data in Section 5.2 rather than assumed. Equation (3) follows from Equation (2) as the kinematic consequence of the same small-sideslip assumption, in which the lateral acceleration in steady-state cornering is the product of the speed and yaw rate. The assumption holds while the lateral acceleration remains within the linear tire range and degrades as the friction limit is approached, which is one reason the identified parameters differ across friction levels. This derivation also anticipates the result of Section 6.2; because Equation (3) makes the predicted lateral acceleration a speed-scaled replica of the predicted yaw rate, the corresponding residual carries no information independent of the yaw-rate residual.

The range of validity of these assumptions should be stated. The small-sideslip approximation holds while the lateral acceleration remains within the linear region of the tire characteristic, which in this corpus covers the great majority of samples; it degrades as the friction limit is approached and fails outright in the transient band between 2 and 5 m s^−1^, where the vehicle has not settled into the steady state that Equation (2) describes. That is precisely the band in which the residual scale is largest, as Section 5.3 shows, and it is the reason a conditional rather than a global normalization is required.

### 4.2. Friction-Conditioned Parameters

The understeer gradient is a tire property and varies with the road-surface friction coefficient [30]; fitting a single global K_us_ across surfaces from μ = 0.35 to μ = 1.00 is therefore mis-specified. We fit the pair (K_us_, τs) separately per friction class. This assumes μ is known, which a production vehicle does not observe directly; the consequences are analyzed in Section 7.5, where the cost of using a single global parameter set bounds the value of online friction estimation.

The identified parameters are not monotonic in μ, and the reason is due to the speed distribution rather than the physics. The understeer gradient is identified by cornering at speed, and the four friction classes do not sample the regime equally: the μ = 0.35 runs spend 51.4% of samples at standstill and never exceed 10 m s^−1^, whereas the μ = 0.65 runs reach 12.9 m s^−1^ at the 95th percentile and spend 6.2% of samples above 10 m s^−1^. The corresponding figures are 45.1% standstill and 4.6% above 10 m s^−1^ at μ = 0.85 and 48.4% and 3.7% at μ = 1.00. K_us_ and τ_s_ are therefore better identified at some friction levels than others, and the ordering of the fitted values should not be read as a physical trend. This is the same mechanism that produces the coverage reversal analyzed in Section 6.7.

### 4.3. Residual and Event-Driven Encoding

The residual is the difference between the measured and predicted yaw rate. In control-engineering terminology this is an analytical redundancy relation [15]; the biological naming does not alter what it computes. After normalization, the standardized residual z(t) is passed through a leaky integrate-and-fire encoder:

*τ_m_ dV*/*dt* = −*V*(*t*) + *z*(*t*);    *s*(*t*) = *sgn*(*V*), *V* ← 0 *if* |*V*| ≥ *V_th_* (4)

We state plainly what this stage is and is not. It comprises a single event-driven leaky integrate-and-fire unit per channel; there are no synaptic weights, no layers, and no learning. Its accumulate-and-reset behavior, followed by a windowed sum, is structurally related to a cumulative-sum statistic with a forgetting factor, and we make no energy or neuromorphic-hardware claim on its behalf. Section 6.5 reports the ablation justifying its retention, as replacing it with the raw standardized magnitude raises the false-alarm rate from 0.76% to 1.51%, which we attribute to its implicit rectification and dead-zone behavior rather than to any spiking property.

### 4.4. Causality

All temporal operators are strictly causal. The moving average over a window of W samples is computed from a trailing cumulative sum C:

*S*(*t*) = [*C*(*t* + 1) − *C*(*max*(0, *t* − *W* + 1))]/[*t* − *max*(0, *t* – *W* + 1) + 1] (5)

Derivatives are evaluated by backward difference. No operator accesses samples at indices greater than the current one. We state this explicitly, as the effect is systematic and in a consistent direction albeit modest in magnitude; the identical pipeline evaluated with a centered filter of the same length reports superior figures that no deployable monitor can realize, and the size of this difference is quantified in Section 6.2.

### 4.5. Operating-Condition Residual Normalization (OCRN)

The residual is strongly heteroscedastic with respect to speed (Table 4). We estimate the conditional mean and standard deviation in B = 18 speed bins from the nominal calibration run and standardize these bin-wise:

*z*(*t*) = [*r*(*t*) − *μ_r_*(*v_b_*(*t*))]/*σ_r_*(*v_b_*(*t*)) (6)

Bin edges are {0, 0.3, 0.6, 1, 1.5, 2, 3, 4, 5, 6, 7, 8.5, 10, 12, 14, 16, 18, 22, ∞} m s^−1^, chosen for their density at low speeds where the residual scale varies most rapidly; bins containing fewer than 40 nominal samples revert to the global statistics. This treatment, formalized as Algorithm 1, is both statistically required and biologically consonant, since peripheral vestibular gain is modulated by the state of self-motion rather than held as fixed [31].

The operator is stated in terms of an operating variable rather than speed. Nothing in Algorithm 1 is specific to longitudinal velocity: the conditioning variable may be any observable quantity that governs the scale of the residual, and the same construction applies to load, temperature, rotational speed, or actuator duty in other monitoring problems. What the operator requires is that the variable be measurable at runtime, that the residual scale varies systematically with it, and that attack-free data covering the operating range be available when commissioned. Where these conditions are held, the operator converts a heteroscedastic residual into an approximately standardized one at constant cost per sample, which is what allows for a single threshold to serve the whole operating envelope. In the present instantiation, the conditioning variable is vehicle speed, chosen because Table 4 shows the residual scale varying by a factor of 158 alongside it.

The construction also has a biological reading. Peripheral vestibular gain is not fixed but is modulated according to the state of self-motion, so that the same afferent channel remains informative across widely different movement regimes. OCRN performs the engineering counterpart of that modulation, and Section 6.5 shows it to be the element of the architecture that carries the transfer, while the more literal implementation of efferent attenuation does not.

The operator is defined in terms of three choices that are stated here explicitly. The operating-condition variable is any quantity observable at runtime whose value governs the scale of the residual; here, it is longitudinal speed, and Section 7.5 discusses what would change under a different choice. The residual distribution is estimated non-parametrically rather than assumed, where within each of the B = 18 bins, the conditional mean and standard deviation are computed from the attack-free calibration data, subject to a minimum count of n = 40 samples, with a fallback to the global statistics when a bin is too sparse and with a variance floor of 10−4 rad s^−1^ to guard against degenerate bins. The normalized residual then enters the decision rule unchanged, through the encoder of Section 4.3 and the causal window of Section 4.4, so that the operator can be inserted into or removed from an existing detector without altering anything downstream. This is what makes the cross-comparison of Section 6.6 possible.

### 4.6. Learned Forward-Model Correction

Biological internal models are acquired rather than analytically derived. We therefore augment the analytic prediction with a learned correction. A ridge regression is fitted on nominal, attack-free residuals only, using 36 features constructed from the command history; lagged steering in 0, 3, 8, 15, 30, 60, and 120 samples interacted with speed under three functional forms, whereas throttle and brake interacted with the speed, steering rate, longitudinal acceleration, and speed polynomials. To exclude information leakage, the correction is fitted under a leave-one-town-out cross-validation: the model applied to any Town-04 scenario is fitted exclusively on Town-01, -02, -03, and -05 data. The reported coefficient of determination is therefore an out-of-town generalization figure.

The correction is a ridge regression. The input is a 36-dimensional feature vector comprising, at seven lags, the steering-speed product, its model-consistent variant, a quadratic speed term, and the steering angle alone, together with speed polynomials, throttle and brake interactions, steering and speed rates, and sign and magnitude terms. The target is the residual of the analytic forward model. The loss is a squared error with an L2 penalty of 10-2, and the training data for each evaluated scenario are drawn from all the scenarios with the other four towns, so that no scenario contributes to the correction applied to itself or to any scenario from its own town.

### 4.7. Maneuver-Dependent Attenuation Gate

The efferent gain-modulation element of the reafference principle is implemented as

(7) $$\gamma (t)=1/(1+\beta_{1}\;|{\overset{\cdot }{\delta }}_{cmd}(t)|+\beta_{2}\;|\delta_{cmd}(t)|)$$

The gated score is the causal moving average of the encoder output multiplied by γ. The coefficients β1 and β2 are treated as decision variables in the optimization rather than fixed a priori. As Section 6.5 reports, both are driven to zero at the identified operating point; the gate is not selected under the multi-objective formulation, and we retain it for completeness and for the analysis in Section 7.2 rather than as a contribution.

### 4.8. Decision Rule

An alarm is raised when the gated score exceeds a threshold set at the p-th percentile of the score distribution of the nominal calibration run. An optional persistence requirement of n consecutive exceedances is available and is included in the search space.

### 4.9. Design-Space Exploration as a Constrained Multi-Objective Problem

The monitor exposes six free parameters: the leaky-integrator time constant, *τ_m_*; the firing threshold, *V_th_*; the causal window length *W*; the calibration percentile, *p*; and the two attenuation coefficients, *β*_1_ and *β*_2_. Selecting these is not a matter of tuning a single scalar. The design is a constrained multi-objective problem in which the detection quality, false-alarm rate, and detection latency trade against one another, and in which feasibility is defined per scenario rather than in aggregate. We state the problem as

*minimize*   {−*F*_1,*mean*_(*θ*), −*F*_1,*worst*_(*θ*), *FAR_mean_*(*θ*), *TTD_mean_*(*θ*)} (8)

*subject to   FAR_s_*(*θ*) ≤ *FAR_max_    for every scenario s* (9)

where *θ* = (*τ_m_*, *V_th_*, *W*, *p*, *β*_1_, *β*_2_). The first two objectives are deliberately separated: averaging detection quality across scenarios conceals the scenarios in which the monitor is blind, and a design that is optimal in the mean is not necessarily feasible in the worst case. The lateral-acceleration channel weight and the persistence requirement are treated as additional discrete decision variables. Equations (8) and (9) define the objective vector and the scenario-wise feasibility condition, respectively.

Because each evaluation requires simulating the full pipeline over all 25 scenarios, the objective functions are expensive rather than analytic. We therefore resolve the design space by exhaustive enumeration over a structured grid of 9600 configurations, giving 240,000 scenario evaluations in total. This is tractable at the present dimensionality and provides an exact reference for the feasible set that a stochastic search could not. Grid boundaries were widened whenever a selected value fell on an edge, and the reported operating point is interior in every dimension. The exhaustive treatment also permits a question that a single optimized solution cannot answer: how the feasible set itself changes with the constraint level. Section 6.2 reports this sweep, and Section 7.1 uses it to separate the contribution of each design component from the contribution of the evaluation protocol.

The per-sample cost of the monitor is constant. The forward model requires one first-order lag update and one division; the encoder requires one membrane update and a comparison; the normalization requires one bin lookup, one subtraction, and one division; and the windowed statistic is maintained by a trailing cumulative sum, so its cost does not grow with the window length. The monitor is therefore has a constant time per sample and is linear in the length of a run, with a memory footprint of one cumulative-sum register, one membrane state per channel, and the stored bin statistics. The design-space exploration scales with the product of the number of configurations, the number of scenarios, and the run length, and is performed offline. Measured inference latency is reported in Section 5.1 with its distribution rather than its mean, as the control period and the inference latency are separate quantities and conflating these overstates the real-time margin.

The enumeration is exhaustive by design. The six decision variables correspondingly take 5, 4, 6, 5, 4, and 4 values, which gives 9600 configurations; each is evaluated on all 25 scenarios, so the search performs 240,000 scenario evaluations. The enumeration is offline and completes in minutes rather than hours on the platform stated in Section 5.1, because the encoder pass is cached across all configurations that share a time constant and firing threshold, and only the windowing, gating, and thresholding are repeated. Enumeration is preferred to a stochastic search because the object of interest in this section is the feasible set rather than the optimum, and only exhaustive evaluation characterizes this set exactly. The risk that such a large search would overfit the evaluation scenarios is real and is quantified in Section 6.9.

## 5. Experimental Setup

### 5.1. Data

Injecting false data into the inertial channel of a vehicle in motion is not something that can be done safely on a public road, and no public dataset contains inertial measurements recorded under an actual injection attack. The evaluation is therefore conducted in simulation, where the injection is controlled and the ground-truth is exact. Telemetry was collected in CARLA [32] under synchronous mode at a fixed step of 0.01 s, with a Tesla Model 3 blueprint under the simulator’s autopilot. Twenty-five scenarios were recorded from five towns with five surface and weather configurations, each for a duration of 90 s. At 100 Hz, this corresponds to a nominal 225,000 samples, of which 224,999 were written to file.

Two cleaning rules were applied. The first ten samples of each scenario were discarded as spawn transients, and samples with absolute lateral acceleration exceeding 50 m s^−2^ were removed as collision or spawn artifacts; the raw maximum exceeds 3.6 × 10^6^ m s^−2^, which is not physical. Together, after removing 361 samples (0.16%)—250 spawn transients and 111 acceleration artifacts—this leaves 224,638. One property of the dataset proved decisive: 46.7% of all samples were recorded below 0.5 m s^−1^, reflecting realistic urban stop-and-go behaviors under traffic-signal control, with per-scenario standstill fractions ranging from 19.7% to 88.3%.

The resulting scenario matrix is given in Table 5.

All experiments in this paper, including the telemetry collection, the design-space enumeration, and the timing measurements, were run on a single workstation: an Intel Core i9-11900K at 3.50 GHz with 16 logical cores and 64 GB of RAM, an NVIDIA GeForce RTX 3080 Laptop GPU used only by the simulator, under Windows 11 Pro (build 26200). The software environment is Python 3.7.9 with NumPy 1.21.6 and SciPy 1.7.3; the Python version is fixed by the simulator as a requirement. The monitor is a reference implementation in Python and NumPy and is not optimized. Per-sample latency was measured with time.perf_counter over the encoder update, the bin lookup, and the standardization, averaged over blocks of 2000 samples and repeated thirty times; the median is 0.380 microseconds, the 95th percentile is 0.403, and the 99.9th percentile is 0.423 against a control period of 10 milliseconds. The figure bounds the cost of the arithmetic on a general-purpose processor in an interpreted language and is not an embedded-target benchmark; what it establishes is that the per-sample cost is constant and small relative to the control period and not that a particular platform achieves it.

### 5.2. Forward-Model System Identification

Four structures were compared on attack-free data above 2 m s^−1^ (114,098 samples). Geometry was fixed at the simulator values; only K_us_ and τ_s_ were fitted by exhaustive search over K_us_ in [0, 0.40] s^2^ m^−1^ at 81 points and τ_s_ in [0, 1.7] s at 12 points.

The outcome of the structure selection is reported in Table 6.

Friction-conditioned parameters were K_us_ = 0.030, 0.055, 0.030, and 0.015 s^2^ m^−1^ with τ_s_ = 1.00, 0.30, 0.40, and 0.10 s for μ = 0.35, 0.65, 0.85, and 1.00, respectively. The negative coefficient of determination of M1 warrants explicit statement: the purely kinematic model predicts the yaw rate less effectively than a constant does, because a normalized steering command is passed to the tangent function as though it were an angle. An efference copy constructed on M1 carries negative information, and no threshold policy can repair this.

**Figure 2:**
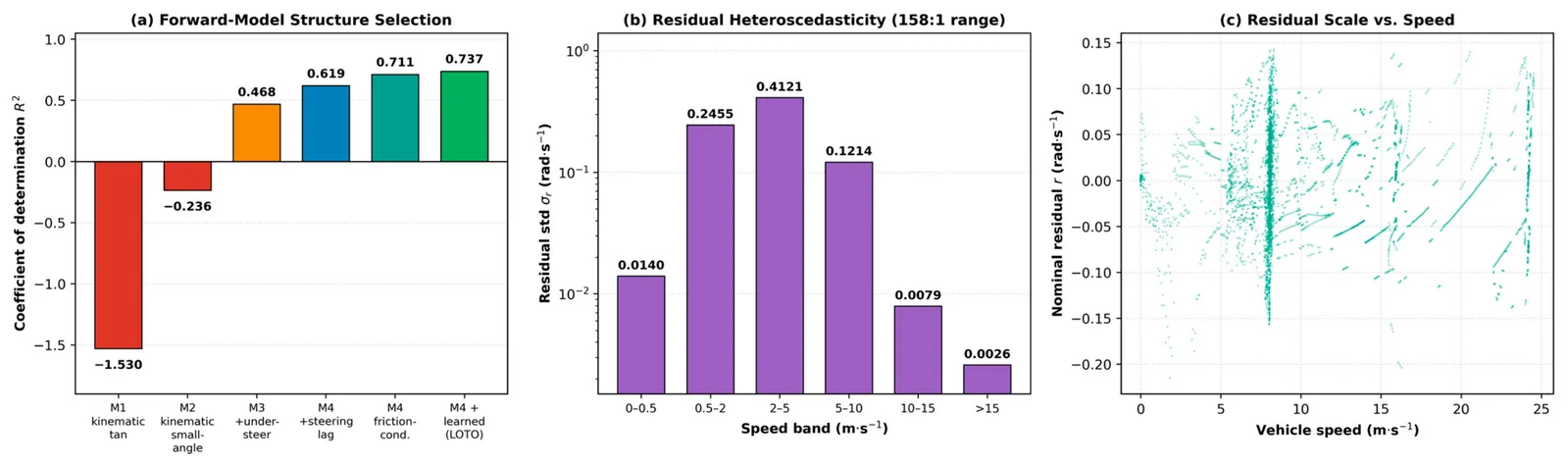
Forward-model identification and residual scale. (**a**) Coefficient of determination for the four candidate structures and two refinements. (**b**) Nominal residual standard deviation by speed band, logarithmic ordinate; the ratio between the worst and best bands is 158:1. (**c**) Nominal residual against vehicle speed for a representative scenario. The band statistics in panel (**b**) are identical to those in Table 4; panel (**a**) is computed on samples above 2 m s^−1^ and panel (**b**) on all samples, which is why the two are not directly comparable.

Figure 2 summarizes the identification results and the residual scale that follows.

The friction-conditioned parameters were identified on the same corpus on which the monitor is subsequently evaluated. To quantify the optimism this introduces, the identification was repeated under a leave-one-town-out protocol, in which the parameters for each friction level are re-estimated from four towns and evaluated on the fifth. Table 7 reports both fits. The re-identified parameters are close to the in-sample values and the optimism in the coefficient of determination is 0.0166 averaged over friction levels, ranging from 0.0005 at the highest friction level to 0.0462 at the lowest, where the speed distribution is least favorable for identification.

Three fault types are covered by the injection classes of Table 2—an oscillatory deviation, a constant bias, and a slow drift—which are all applied abruptly and placed identically across scenarios so that the classes are comparable. Three further types are not covered, and we state this rather than leave it implicit: multiplicative scaling errors, intermittent faults with stochastic onset, and additive sensor noise. Their omission narrows the scope of the detectability figures reported here to additive, persistent deviations of the yaw-rate channel, and Section 7.5 identifies their inclusion as required future work.

### 5.3. Residual Heteroscedasticity

Standstill is not the difficult regime: with both prediction and measurement near zero, the residual is small. The difficulty lies in the 0.5–5 m s^−1^ launch-and-brake band, where the steady-state model assumption is least valid. The model predicts the yaw rate that a given steering command should produce once the vehicle has settled into it. During pull-away and braking, the vehicle has not settled, so the prediction is systematically wrong and the residual is large for reasons that have nothing to do with an attack. A single global standardization constant is therefore simultaneously two orders of magnitude too large above 15 m s^−1^ and too small within the transient band.

The ratio quoted throughout is the ratio of residual standard deviations between the extreme speed bands of the analytic forward model reported in Table 4; 0.4121 rad s^−1^ in the 2–5 m s^−1^ band, estimated from 13,609 samples, against 0.0026 rad s^−1^ above 15 m s^−1^, estimated from 5988 samples, for a ratio of 158.5, quoted throughout as a factor of 158. Bootstrap 95% confidence intervals on each band standard deviation are given in Table 4 and are narrow relative to the differences between bands. The corresponding ratio under the learned correction is substantially smaller, which is itself evidence that the correction removes a substantial part of the heteroscedasticity rather than merely reducing the mean error.

### 5.4. Calibration Protocol

The decision threshold is calibrated on an attack-free run recorded under the same road-surface conditions rather than on the opening seconds of the deployment run. This reflects how such a monitor is commissioned in practice: nominal data is collected during validation, the residual statistics and the threshold are fixed, and the monitor is then deployed. Section 6.4 quantifies the penalty incurred by the alternative startup-window protocol.

### 5.5. Baselines and Statistical Protocol

Two baselines are evaluated: a windowed chi-squared test on the standardized residual pair, and a cumulative-sum procedure on the residual magnitude with drift factor k. Each baseline is searched exhaustively over the whole of its own design space: a window length over ten values; a threshold percentile over nine; persistence over six; and, for the cumulative-sum procedure, a drift factor over eight. Each is additionally evaluated under two normalization conventions, a single global standard deviation and the proposed operator, so that the effect of the detection rule can be separated from the effect of the normalization. All configurations are evaluated under identical calibration, causality, and metric conventions. The resulting budgets are stated in Section 6.6. The proposed monitor has more free parameters and therefore a larger grid; what is equalized is the exhaustive coverage of each method design space rather than the number of evaluations, and the controlled comparison of Section 6.6 crosses the detection rule with the normalization so that neither factor is confounded with search effort. Reporting a single hand-selected operating point for a baseline against an exhaustively searched proposal does not constitute a comparison, and we therefore do not do so.

Per-scenario metrics are treated as 25 paired observations. Method comparisons employ the Wilcoxon signed-rank test [33] with Holm correction [34]; effect sizes are reported as Cliff deltas [35]; and pooled metrics carry bootstrap 95% confidence intervals. We acknowledge that the hyperparameters are selected on the same 25 scenarios subsequently reported. This is mitigated by the interiority of the selected operating point within the search grid and by the flatness of the performance surface in its neighborhood, but it is not eliminated.

### 5.6. Detectability Floor

A monitor cannot detect an injection smaller than the noise it must reject. For a residual of scale σr and a one-sided test at false-alarm rate α, the minimum detectable bias [6] is

*δ_min_* = *k σ_r_* (10)

where k is determined by α. A target of α = 1% corresponds to k ≈ 2.33, and k = 3 is the conventional conservative choice, consistent with the 0.76% false-alarm rate measured at the identified operating point. We therefore report recall in two forms. The first is unrestricted (k = 0), where each labeled attack sample is counted, including those whose injected magnitude falls below any achievable floor; this is the conservative figure. The second as floor-restricted (k = 3), where recall is computed over injected samples clearing the floor, and the coverage, defined as the proportion of injected samples clearing it, is reported alongside every such figure. The parameter k is swept over {0, 1, 2, 3}, and no single value is privileged.

The floor is defined as δ_min_(x) = k σ_r_(x), where x denotes the operating point, σ_r_ is the bin-conditional residual standard deviation estimated by the operator at that operating point, and k fixes the nominal exceedance level. The dependence on speed enters directly through σ_r_. The dependence on friction is indirect, through the effect of friction on forward-model error, and Section 6.7 shows that this indirection matters: the floor tracks the residual scale closely and the friction level only loosely. The definition is a statement about scale and not a probabilistic guarantee, and it presumes only that σ_r_ is estimated on attack-free data representative of the operating point.

The choice of k warrants a caution. Under a Gaussian assumption, a multiplier of 2.33 corresponds to a one-sided exceedance probability of 1%, and three is the conventional conservative choice. The assumption does not hold here. The standardized residual has excess kurtosis of 135.0 and skewness of −0.69, and the observed exceedance rates are 2.73% beyond 2.33 standard deviations against a nominal 1%, and 1.45% beyond three against a nominal 0.13%. The multiplier must therefore be read as a scale convention rather than as a calibrated probability, which is why the full sweep over k is reported in Section 6.2 instead of a single value being presented as derived. A deployment should select k from its own operating requirement.

The injection magnitudes were chosen to span the detectability range rather than to represent a specific threat model, and we state this as a limitation. To give them an external referent, each class can be expressed as the lateral acceleration it would induce. At the corpus mean speed of 4.09 m s^−1^, the three classes correspond to 1.64, 2.45, and 2.62 m s^−2^; at the 95th-percentile speed of 8.38 m s^−1^, they correspond to 3.35, 5.03, and 5.36 m s^−2^. All three therefore exceed, at the higher speeds present in the corpus, the 3.0 m s^−2^ lateral-acceleration ceiling that UN Regulation No. 79 places on automatically commanded steering functions in Category B1. We do not claim that this constitutes a threat model, but it establishes that the magnitudes are of a safety-relevant order rather than arbitrary.

Recall and coverage answer different questions and are reported together for that reason. Recall is the proportion of injected samples that raise an alarm; coverage is the proportion whose magnitude exceeds the floor at the operating point at which they occur. Recall alone is insufficient because its denominator includes injections the monitor cannot detect in principle, so a monitor evaluated on a corpus containing many small injections appears worse than the same monitor evaluated on a corpus containing large ones, with no difference in the monitor itself. Reporting coverage alongside recall makes the composition of that denominator explicit, and the sweep over k in Section 6.2 allows readers to recover either convention.

All figures in this paper are generated by scripts that read the same result files that produce the tables, such that no figure is computed independently of the table it accompanies. Where the panels of a figure use different sample restrictions, the restriction is stated in the caption of that figure. The remaining panels plot the rows of the corresponding table directly, without recomputation.

## 6. Results

The identified operating point is τ_m_ = 0.20 s, Vth = 0.30, W = 10 samples (100 ms), *p* = 99.0, β_1_ = β_2_ = 0, and lateral-acceleration weight w_a_ = 0.

### 6.1. Detection Performance

The pooled and per-scenario figures are given in Table 8.

We state the recall in prose because it is the figure most frequently omitted in the literature: when counting all labeled attack samples, the monitor misses 23.65% of them; when counting only injections above the 3σ floor, it misses 6.01%. The 19.9% of samples excluded by the floor carry injected magnitudes averaging approximately one fifth of those retained.

**Figure 3:**
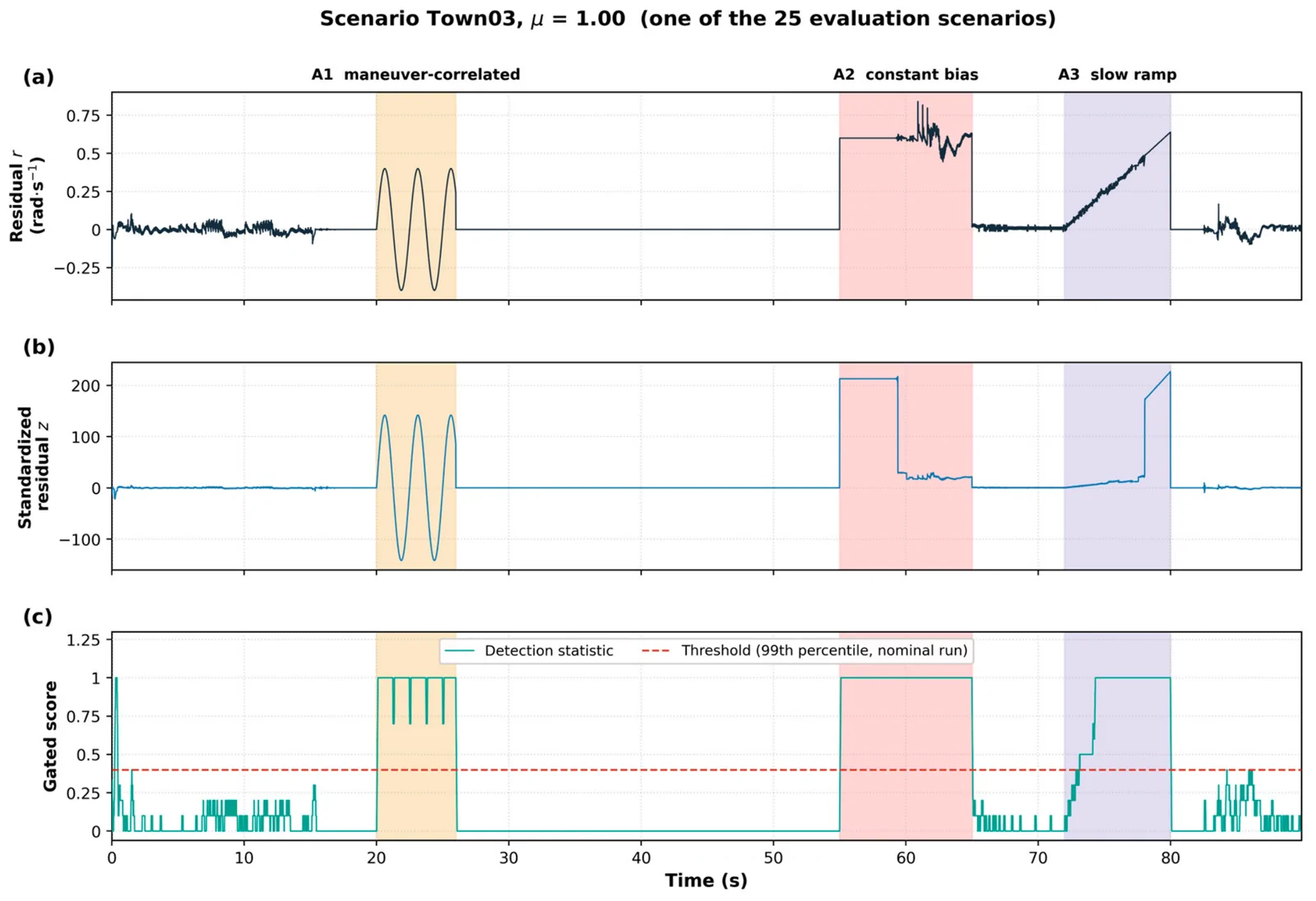
Monitor operation on a representative scenario. (**a**): Raw residual. (**b**): Residual standardized by the OCRN operator. (**c**): Gated detection statistic against the threshold calibrated on the nominal run. Shaded intervals mark the three injection windows.

Figure 3 shows the operation of the monitor on a representative scenario.

Scenario-level bootstrap 95% confidence intervals, obtained from 10,000 resamples of the 25 scenarios, are as follows. At k = 0: precision 97.34% [96.78, 97.81]; recall 76.35% [67.09, 84.42]; F_1_ 85.57% [79.32, 90.50]; and false-alarm rate 0.76% [0.63, 0.90]. At k = 3: precision 97.30% [96.73, 97.78]; recall 93.99% [91.16, 96.30]; F_1_ 95.62% [94.03, 96.87]; and false-alarm rate 0.76% [0.63, 0.90]. The width of the recall interval at k = 0 is itself informative, as it is the scenario heterogeneity that the detectability floor is introduced to expose, and it narrows by more than a factor of three once the floor is applied.

### 6.2. Detectability Floor and Channel Selection

Table 9 reports the sweep.

The false-alarm rate is exactly invariant with respect to k, because it is computed on attack-free samples which the floor does not touch. Precision is very nearly but not exactly invariant, drifting by 0.04 percentage points across the sweep: raising k removes injected samples from the attack class, and the small number of these that had been correctly alarmed migrate out of the true-positive count.

Table 10 reports this validation.

The channel comparison is given in Table 11.

Combining the channels is inferior to the yaw-rate channel alone, and the optimizer assigns zero weight to the acceleration channel across the entire search grid. The explanation is structural rather than empirical; under Equation (3), the acceleration residual is a speed-scaled replica of the yaw residual carrying additional sensor noise, together with additional model error from the road bank and grade, neither of which the model represents. It contributes noise without contributing independent information.

**Figure 4:**
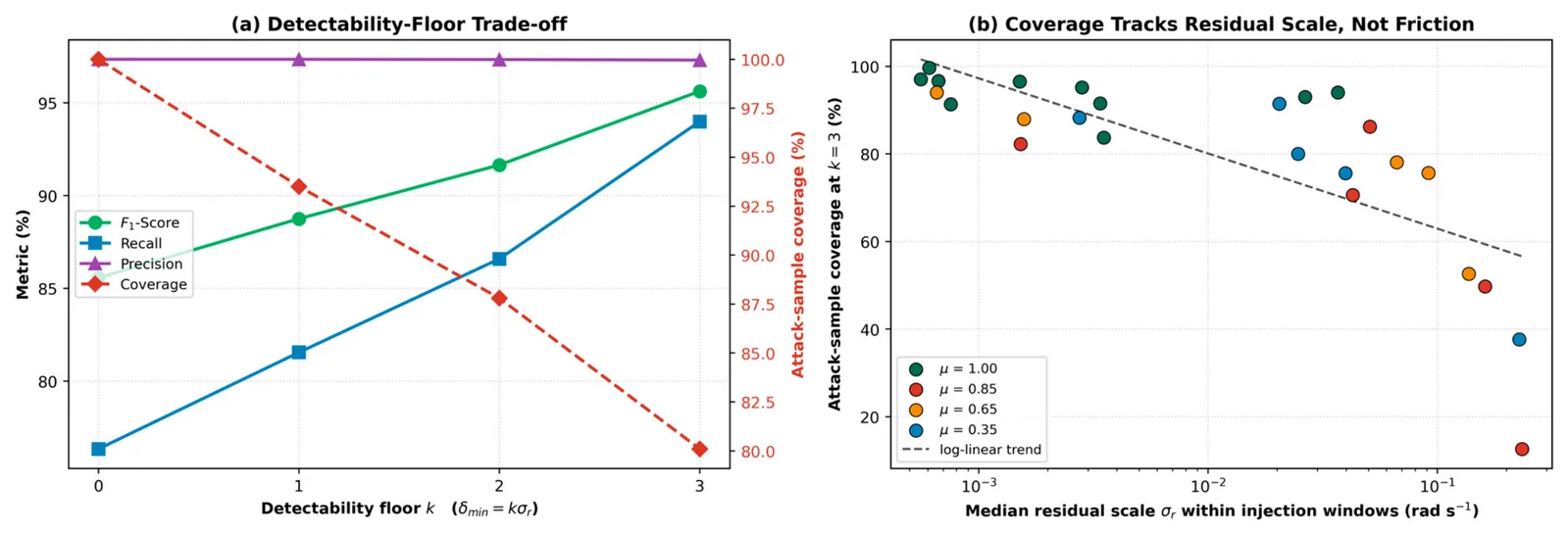
Detectability floor. (**a**) Precision, recall, and F_1_ against the floor parameter k, with attack-sample coverage on the secondary ordinate. (**b**) Coverage at k = 3 for each of the 25 scenarios against the median residual scale within its injection windows, colored by friction level. Coverage tracks the residual scale (Pearson r = −0.946 on the linear scale); the friction levels are interleaved rather than ordered, which is the basis for restatement in Section 7.3.

Figure 4 shows the trade-off and the mechanism behind the per-scenario variation.

The cost of causality can now be stated numerically. Replacing the causal trailing-window statistic by a centered window of identical length, and with every other element unchanged, moves the reported figures at k = 3 from 97.30% to 97.80% precision, 93.99% to 94.66% recall, 95.62% to 96.20% F_1_, and 0.76% to 0.62% false-alarm rate. The corresponding F_1_ difference at k = 0 is 0.43 points. The effect is therefore systematic and always in the direction of flattering the non-causal variant, but it is of the order of half an F_1_ point rather than large.

The floor is not merely a restatement of the tuning. Grouping injected samples by the ratio of injection magnitude to the local residual standard deviation, and computing recall within each group, without reference to the floor, gives the monotone relation of Table 10. Recall rises from 1.64% below a ratio of one to 99.61% above ten, and the transition occurs between ratios of three and five, which is where the floor is placed. The injected samples excluded at k = 3 also differ in amplitude from those retained; their mean absolute deviation is 0.266 rad s^−1^ against 0.460 rad s^−1^ for the retained samples, with medians of 0.219 and 0.598 rad s^−1^, respectively.

### 6.3. Attack-Class Behavior

Recall and mean time-to-detect are reported by attack class in Table 12.

The constant-bias case is detected essentially completely and is unaffected by the floor since its injected magnitude exceeds three residual standard deviations in every scenario. The shortfalls for A1 and A3 are almost entirely accounted for by sub-floor samples: A1 crosses zero five times within its window, and A3 begins at zero and requires approximately 2.7 s to exceed the residual scale. This is consistent with the established difficulty of detecting incipient sensor faults, whose magnitude grows slowly from zero [36].

### 6.4. Calibration-Transfer Penalty

The effect of the calibration protocol is quantified in Table 13.

The startup-window protocol, common in the literature, is not merely suboptimal: it produces a four-fold higher false-alarm rate while the detection statistic itself is unchanged.

### 6.5. Component Ablation

Three results in this table are unfavorable to the framing that originally motivated the work, and we report them accordingly.

First, OCRN dominates every other component. Its removal costs 21.8 F_1_ points and reduces the worst-scenario F_1_ to zero; at least one scenario becomes entirely blind. No other component contributes more than 1.5 points. The principal finding of this paper is therefore not a detection rule but a normalization.

Second, the maneuver-dependent attenuation gate is not selected. Enabling it with β_1_ = 1.8 reduces F_1_ from 95.62% to 95.39%, raises the false-alarm rate from 0.76% to 1.02%, and reduces the worst-scenario F_1_ from 79.63% to 76.23%. The variant with β_2_ = 0.8 attains a marginally higher pooled F_1_ but at nearly twice the false-alarm rate and with lower precision. The optimizer consequently sets both coefficients to zero under the multi-objective formulation of Section 4.9, in which the false-alarm rate is itself an objective. The element of the architecture that is most explicitly biological is the one that does not pay for itself.

The precise statement is that the gate is not selected rather than that it is without effect. Searched jointly with the other parameters over the full grid, the best gate-enabled configuration attains a pooled F_1_ of 96.62% against 95.62% for the best gate-free configuration but at a mean false-alarm rate of 1.20% against 0.76%. Holding every other parameter at the identified operating point and enabling the gate alone gives 96.08% F_1_ at a 1.40% false-alarm rate. Neither configuration dominates the other in the objective vector of Equation (8), and no configuration in the grid dominates the operating point reported here. The gate therefore gains at most one F_1_ point at roughly twice the false-alarm rate, which is an order of magnitude below the 21.8 points attributable to the operator, and it does so while creating the exposure window analyzed in Section 7.2.

Third, the learned correction improves robustness rather than accuracy. It leaves the pooled F_1_ essentially unchanged while reducing the false-alarm rate and raising the worst-scenario F_1_ by 14.4 points, from 65.24% to 79.63%. Its benefit lies in the tail of the scenario distribution not in the mean.

**Figure 5:**
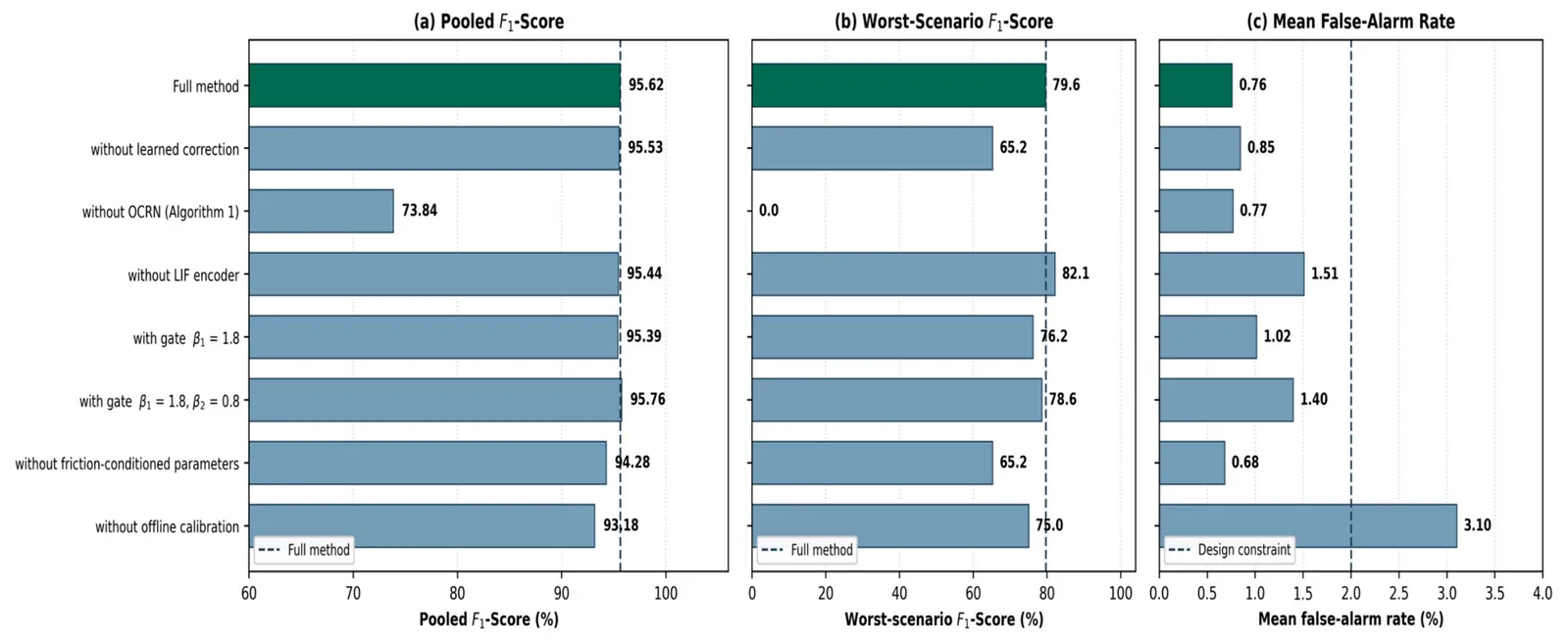
Component ablation at k = 3. (**a**) Pooled F_1_. (**b**) Worst-scenario F_1_. (**c**) Mean false-alarm rate against the 2% design constraint. Removing the OCRN operator is the only modification producing a large effect.

Figure 5 presents the same ablation graphically.

The component ablation is reported in Table 14.

### 6.6. Detection Rule Against Normalization

Two questions are separated here. The first is whether the detection rule matters; the second is whether the normalization matters. Table 15 crosses the two factors. Each detector is searched exhaustively over its own design space under each normalization, subject to the same mean false-alarm constraint. Under a single global standard deviation, the windowed chi-squared test attains a scenario-mean F_1_ of 76.18 and the proposed encoder 79.86. Under the operator, they attain 94.74 and 94.83. The cumulative-sum procedure fails the false-alarm constraint under either normalization. Changing the detection rule therefore moves the result by 0.09 F_1_ points, while changing the normalization moves it by between 15 and 19 points.

The paired tests over the 25 scenarios confirm this reading. Against the cumulative-sum procedure the proposed monitor is superior by 45.98 F_1_ points, with a Wilcoxon signed-rank statistic of 0.0, a Holm-corrected *p*-value of 1.19 × 10^−7^ and Cliff delta of +1.000. Against the windowed chi-squared test operating on the same normalized residual the difference is −0.16 points, with a statistic of 127.0, a Holm-corrected *p*-value of 0.353 and Cliff delta of −0.018, which is a negligible effect size. We report this as it stands. It is the strongest available evidence for the claim this paper actually makes, namely that the contribution lies in the conditional normalization and not in the architecture of the detector that consumes it.

The two grid sizes quoted in this paper refer to different searches and are not in conflict. The 9600 configurations of Section 4.9 form the design-space enumeration that selects the operating point; it varies the encoder time constant, the firing threshold, the window length, the calibration percentile, and both gate coefficients, and holds the persistence requirement at one. The 10,800 configurations of Table 15 are a different enumeration, constructed so that the proposed detector and the two baselines are searched over comparable axes; the gate coefficients are held at zero, since neither baseline has a gate, and the window length, percentile, and persistence requirements are varied over the same ten, nine, and six values used for the baselines. The first search answers which configuration to deploy; the second answers whether the detection rule matters once the normalization is fixed.

The sample-level false-alarm rate reported in the preceding tables is not the quantity an integrator would specify. Expressed at the event level, as contiguous alarm blocks rather than samples, the identified operating point produces 309 alarm events per hour. Stratified by speed, the rate is 1.21% at standstill, 0.34% between 0.5 and 5 m s^−1^, and 0.45% between 5 and 10 m s^−1^ and 0.26% above 10 m s^−1^, so the alarms are concentrated where the vehicle is not moving. Table 16 gives the trade-off obtained by raising the persistence requirement, which is n = 1 at the identified operating point.

### 6.7. Per-Scenario Distribution and Friction Dependence

Across all 25 scenarios, the mean F_1_ at k = 3 is 94.58% with a minimum of 79.63%, and the mean false-alarm rate is 0.76% with a maximum of 1.55%.

The five weakest scenarios are listed in Table 17.

Ordered by friction rather than by coverage, the mean coverage at k = 3 is 93.9% at μ = 1.00, 60.3% at μ = 0.85, 77.7% at μ = 0.65, and 74.6% at μ = 0.35. The ordering is not monotone, and the μ = 0.85 group is the weakest in the set. This is not a defect of the data but a consequence of the mechanism, and Table 18 makes this visible. The coverage tracks the residual scale within the injection windows almost exactly, with a correlation of −0.946 across all 25 scenarios, while its correlation with the friction level is only +0.289. Within the μ = 0.85 group, the coverage spread is 12.7% to 86.2%, and the two scenarios that depress the mean—Town03 and Town05—are those with the largest residual scale, 0.162 and 0.234 against a group median of 0.051, and the highest occupancy of the 2 to 5 m s^−1^ transient band in which the steady-state model assumption is least valid. The correct statement is therefore that the floor scales with forward-model error, which is correlated with, but not determined by, tire–road friction.

**Figure 6:**
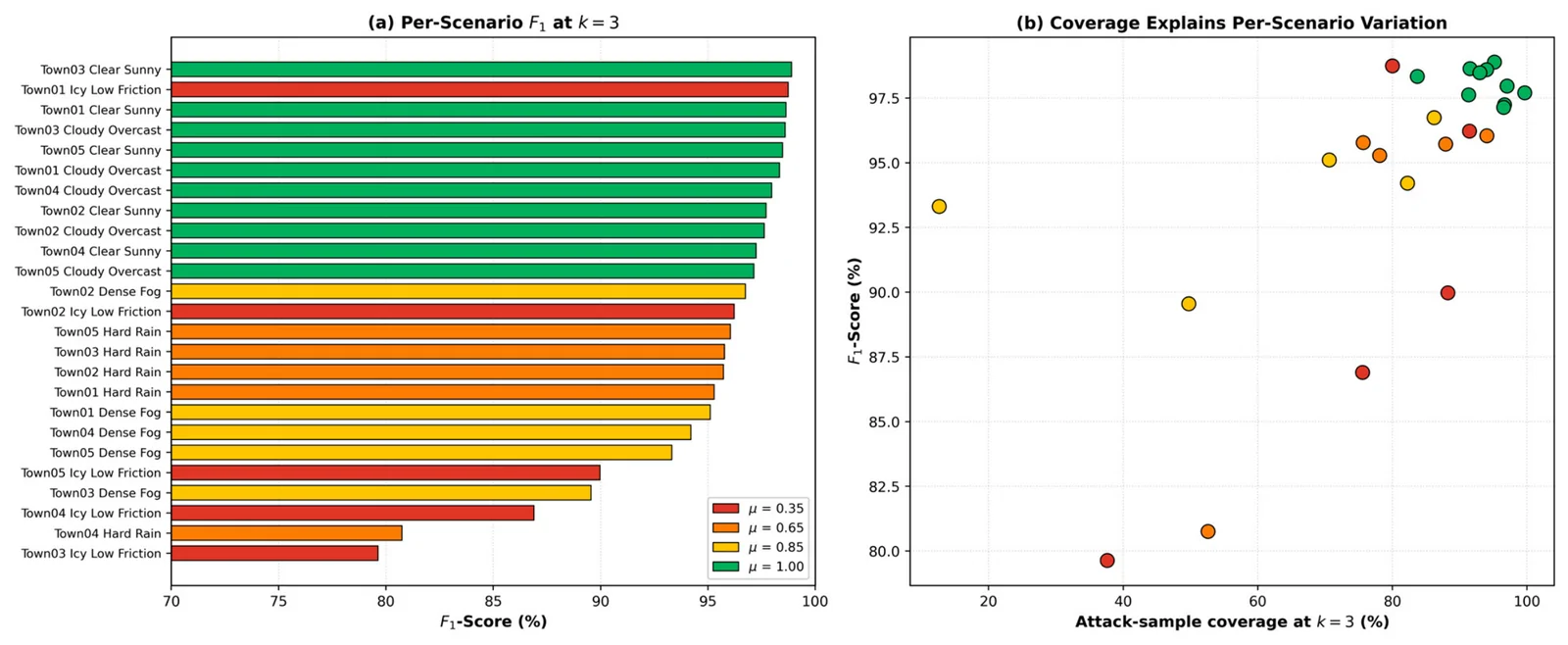
Per-scenario behavior at k = 3. (**a**) F_1_ for all 25 scenarios, colored by friction coefficient. (**b**) Attack-sample coverage against F_1_, showing that coverage accounts for most of the between-scenario variation.

Figure 6 shows the per-scenario distribution and the relation between coverage and performance.

### 6.8. Cross-Location Generalization

Because the learned correction is fitted as leave-one-town-out, the results can be reported for each held-out town separately rather than only in aggregate. Table 19 presents these results. F_1_ ranges from 93.06% on Town04 to 97.35% on Town01, precision from 96.02% to 98.34% and the false-alarm rate from 0.48% to 1.01%. The spread of 4.3 F_1_ points across unseen towns is the appropriate measure of how far the identified configuration transfers geographically, and it is smaller than the spread across friction conditions within any single town.

### 6.9. Selection Bias and Inference Cost

The detector hyperparameters were selected on the same scenarios on which the results are reported. Rather than argue that the resulting bias is small, we measure it. Under a nested leave-one-town-out procedure, the configuration is selected by an exhaustive search across four towns and then evaluated on the fifth, which has not influenced it. Table 20 provides the outcomes. The unbiased estimate is 95.29% precision, 94.87% recall, 95.08% F_1_, and a 1.37% false-alarm rate, against 97.30%, 93.99%, 95.62%, and 0.76% for the single selections reported in the main tables. The selection bias in F_1_ is therefore 0.54 points. Four of the five outer folds selected an identical configuration, which indicates that the design surface is flat in the neighborhood of the operating point rather than that the selection has fitted the evaluation data. The bias in the false-alarm rate is proportionally larger, from 0.76% to 1.37%, and we note this explicitly: the single selection optimizes the false-alarm rate, as well as the detection quality.

Inference cost is reported in Section 5.1: the per-sample latency of the reference implementation has a median of 0.380 microseconds, a 95th percentile of 0.403 and a 99.9th percentile of 0.423 against a control period of 10 ms. These figures are distinct from the time-to-detect values of Table 12, which measure how long an injection must persist before an alarm is raised. The quantity of interest is the constant per-sample cost rather than the absolute latency, which an optimized embedded implementation would reduce further.

## 7. Discussion

### 7.1. What Determines Reported Performance

The single largest contributor in Table 14 is neither the detection rule, the encoder, nor the biological gate. It is the recognition that the residual scale varies by two orders of magnitude across the operating envelope, together with the corresponding decision to standardize conditionally on speed. This is worth 21.8 F_1_ points; every other component contributes at most 1.5, and one contributes negatively.

We take this to carry a general implication. Detection figures reported for vehicle integrity monitors are dominated by protocol choices (causality, calibration, normalization, and which faults are counted as detectable) rather than by the detection statistic itself. Two papers proposing different detectors, evaluated under different conventions, are not comparable, and the difference between their conventions will typically exceed the difference between their algorithms. We would encourage the reporting of the dimensions listed in Table 1 as a minimum.

We would extend this observation to bio-inspired algorithm design more broadly. The component that motivated this architecture, and that carries the clearest biological interpretation, is the one that did not pay for itself; the component that mattered most is a statistical treatment with a biological analog but no biological necessity. A design methodology that evaluates biologically motivated mechanisms only in aggregate, and only against baselines tuned with a smaller budget, cannot distinguish between these two cases. We therefore see the ablation-with-equal-budget protocol used here not as an accessory to the result but as a prerequisite for claiming that any biologically inspired component contributes anything at all.

The comparison of Section 6.6 requires one qualification and yields one conclusion. The qualification is that the proposed monitor has more free parameters than either baseline, so the number of configurations evaluated is not equal and cannot be made so; what is equalized is the exhaustive coverage of each design space, and the cross-design separates the detection rule from the normalization so that neither is confounded with search effort. The conclusion is that under this protocol the detection rule is not what distinguishes the proposed monitor. A windowed chi-squared test operating on the same conditionally normalized residual is statistically indistinguishable from it. We regard this as the central result rather than as an inconvenience because it isolates the contribution: the operator accounts for between 15 and 19 F_1_ points depending on the detector, while the detector accounts for 0.09.

### 7.2. The Attenuation–Exposure Trade-Off

The maneuver-dependent gate reduces sensitivity when the steering command is changing rapidly. The attack class A1 is defined as maneuver-correlated, so the gate reduces sensitivity precisely within the interval that A1 is designed to occupy. Our measurements show the cost is real, as at the identified operating point, enabling the gate with the coefficients of Table 14 raises the false-alarm rate from 0.76% to 1.02% while reducing both pooled and worst-scenario F_1_, and the settings that do raise F_1_ do so only by paying in false alarms.

We attribute this to the interaction with OCRN. The gate was conceived to suppress false alarms induced by maneuvers; but once the residual is standardized conditionally on speed, the maneuver-induced component of the residual is already largely accounted for, and further attenuation removes signal without removing noise. The two mechanisms address the same phenomenon, and the statistical treatment is the more effective of the two.

The finding generalizes beyond this implementation. Command-conditioned gating, including the maneuver-dependent thresholding used in production vehicle diagnostics [16], creates an exposure window that an adversary able to observe or infer the steering command can exploit by timing injections to intervals of low gain. Where the normalization already handles the underlying heteroscedasticity, this exposure is purchased for no detection benefit.

The gate was introduced to test a specific hypothesis: the residual is least trustworthy during rapid steering, such that the detection statistic should be attenuated there in the way that efferent signals attenuate vestibular afferents during self-generated movement. The ablation shows the hypothesis to be correct in substance but redundant in effect. Rapid steering is concentrated in the low-speed transient band, and OCRN already inflates the local standard deviation in that band, which already attenuates the standardized residual there. Once the residual is conditionally normalized the gate re-applies an attenuation that has been applied, and the optimizer removes it. We report this as a result about the relationship between the two mechanisms rather than as a failed component: it identifies which of two biologically motivated elements is doing the work.

### 7.3. A Condition-Dependent Detectability Limit

Section 6.7 establishes a relation we consider the most operationally significant result reported here: the minimum detectable injection magnitude scales with the forward-model residual. That residual is correlated with tire–road friction because a low grip degrades the model, although the correlation is loose and the friction coefficient is not the mechanism. Across the 25 scenarios, the coverage correlates with the residual scale at −0.946 and with friction at only +0.289, and the μ = 0.85 group shows the proxy failing outright. On the highest-friction surfaces the monitor clears the floor for 93.9% of injections; in the weakest scenario, which is not the lowest-friction one, it clears it for 12.7%. The practical consequence is that a detectability specification must be expressed as a function of the residual scale at the operating point, which is measurable at runtime by the same operator that performs the normalization, rather than as a function of a friction estimate that the vehicle may not have. We suggest this is the appropriate form for a quantitative impact rating under ISO/SAE 21434.

### 7.4. On the Biological Framing

We have been deliberate in separating those parts of the reafference analogy that carry technical weight from those that do not. The subtraction of a command-driven prediction from a measurement is model-based residual generation and is not novel; designating the prediction a corollary discharge does not alter what it computes, and we do not claim otherwise.

Two elements did carry weight. First, that internal models are acquired rather than derived [10]: the learned correction, fitted on nominal experience, raised worst-case robustness by 14.4 points over a purely analytic model. Second, that peripheral gain is state-modulated: OCRN is the direct engineering counterpart, and it is by a wide margin the largest single contributor in the paper.

The element that did not carry weight is the one that appears most explicitly biological. The maneuver-dependent gate, modeled on efferent attenuation of vestibular afferents during self-generated movement [14], was not selected: the optimizer set both coefficients to zero, and the settings that improve F_1_ do so only at roughly twice the false-alarm rate. We report this because bio-inspired work in this area rarely states which parts of the analogy paid for themselves, and because the resulting picture, that the useful transfer was statistical rather than architectural, is itself informative.

### 7.5. Limitations

Open-loop evaluation. Injections do not propagate through the control loop. Consequences for vehicle trajectory are neither measured nor claimed; closed-loop evaluation is defined for the next study.

Friction assumed as known. The model parameters are indexed by the true friction coefficient, which a production vehicle does not observe directly. Using a single global parameter set costs 1.3 F_1_ points in the mean but 14.4 points in the worst-case scenario, which bounds the value of online friction estimation without substituting for it.

Hyperparameter selection on the evaluation set. While mitigated by the interiority of the selected operating point and the flatness of the performance surface around it, this is not eliminated; an independently collected second dataset is required.

Non-adaptive adversary. The attack classes are canonical injection shapes and not an optimizing opponent. Section 7.2 identifies the specific structure such an opponent could exploit.

Simulator fidelity. Friction is varied through the tire friction parameter of the wheel physics control. We claim variation in the friction coefficient and nothing further; no claim is made regarding ice or aquaplaning physics, which the tire model does not represent.

A single vehicle and single driving policy. Residual statistics depend on both the vehicle platform and the autopilot policy; generalization across other platforms and policies is untested.

Four limitations of the present evaluation should be stated explicitly. First, the simulated inertial channel is noiseless; the sensor model used carries no stochastic noise, and for samples at which the vehicle is at rest, the yaw-rate channel has a standard deviation of 4.2 × 10^−4^ rad s^−1^, which is the numerical residue of the physics solver rather than a sensor-noise floor. The residual scale reported here is therefore dominated by forward-model error, and on a real unit, the floor would be the larger of the model-error and sensor-noise contributions. Second, the vehicle and tire parameters are those of the simulator physics model, which approximates rather than reproduces the corresponding production vehicle, and no validation against instrumented vehicle data has been performed. The absolute floors are properties of this simulator; the structural findings, which follow from the heteroscedasticity of a model residual and from evaluation conventions, are not contingent on the friction model being quantitatively faithful. Third, the evaluation is open-loop throughout. Fourth, the detector hyperparameters were selected based on the evaluation scenarios; the resulting bias has been measured at 0.54 F_1_ points in Section 6.9, but an independent corpus remains the appropriate confirmatory test, and one spanning five vehicle classes and six standardized maneuvers is under collection.

It is worth separating what we expect to transfer from what we do not. The operator itself and the protocol conclusions rest on residual heteroscedasticity and on evaluation conventions, neither of which is specific to this vehicle or this simulator, and we expect both to hold elsewhere. The bin edges, the identified forward-model parameters, and the numerical detectability floors are properties of this platform and this policy, and would require re-identification on any others. A monitor transferred to a different vehicle would therefore need its calibration repeated but not its design revisited.

## 8. Conclusions

We transferred the reafference principle to the problem of inertial-channel integrity in automated vehicles, introduced the Operating-Condition Residual Normalization (OCRN) operator stated in Algorithm 1, and subjected the results to the characterization that a safety-relevant monitor requires.

The monitor attained 97.34% precision, 76.35% recall, and 85.57% F_1_ at a 0.76% false-alarm rate when every labeled injected sample was counted, and 97.30% precision, 93.99% recall, and 95.62% F_1_ at the same false-alarm rate when recall is restricted to injections above the 3σ minimum-detectable-fault threshold, which covers 80.1% of injected samples. These figures and the coverage separating them are reported together throughout, because reporting either in isolation would be misleading.

Three findings generalize beyond this implementation. First, residual heteroscedasticity dominated, as the yaw-rate residual scale varied by a factor of 158 across the speed envelope, and the operator, which conditions the standardization on an observable operating point rather than on a single global constant, contributed 21.8 F_1_ points, exceeding the combined contribution of the detection statistic, encoder, and biological gate. Second, the evaluation protocol dominated the reported performance; causal versus centered filtering, startup versus offline calibration, and the presence or absence of a stated detectability floor together moved the reported F_1_ by more than fourteen points on identical data with an identical detector, such that comparisons across papers not stating these conventions are not interpretable. Third, the detectability limit was condition-dependent and adverse: the floor scaled with the forward-model error, which was correlated with but not determined by tire–road friction, such that a detectability specification must be expressed as a function of the residual scale at the operating point rather than of the friction coefficient. We suggest this is the appropriate form for a quantitative impact rating under ISO/SAE 21434 [4].

Although OCRN is instantiated here on vehicle speed, nothing in Algorithm 1 is specific to longitudinal velocity. The operator applies wherever an observable operating variable governs the scale of a model residual, which is the common case in condition monitoring, and it can therefore transfer to load, temperature, or rotational speed in other diagnostic settings at the same constant cost per sample.

We also report what did not work. The lateral-acceleration channel was redundant under the steady-state model relation and was discarded by the optimizer. The maneuver-dependent attenuation gate, the most explicitly biological element of the architecture, was not selected once OCRN was in place; its best setting gained at most one F_1_ point at roughly twice the false-alarm rate, and the multi-objective search set both coefficients to zero. The learned forward-model correction improved robustness but not accuracy, reducing the false-alarm rate and raising the worst-scenario F_1_ by 14.4 points.

A consequence of this finding deserves to be stated plainly rather than left for the reader to infer. Under the cross-comparison of Section 6.6, a windowed chi-squared test operating on the same conditionally normalized residual was statistically indistinguishable from the monitor proposed here. We report this because it isolates the contribution rather than diminishing it; what the architecture supplies is the operator, and the detection rule that consumes its output may be chosen on other grounds. Future work follows directly. Closed-loop propagation is required to connect detection to vehicle consequence; online friction estimation would remove the assumption that the friction coefficient is known; and evaluation against an adversary that optimizes against the residual structure remains open.

## Figures and Tables

**Table 1:** Comparison on evaluation-protocol dimensions.

Aspect	χ^2^/CUSUM	Wang & Li [21]	Learnable-Membrane SNN	This Work
Causal (no look-ahead)	Yes	N/A (offline)	Partly	Yes, enforced
Forward model of vehicle motion	Assumed	No	No	Identified
Inertial channel treated as	Monitored	Trusted reference	Monitored	Monitored
Heteroscedasticity handled	No	N/A	No	OCRN
Detectability floor stated	No	No	No	Derived and swept
Recall reported	Varies	Yes	Yes	Always
Per-scenario worst case reported	No	No	No	Yes
Baseline search budget	—	Equal	Unstated	Equal
Closed-loop propagation	Some	No	No	No (stated)

**Table 2:** Injection profiles applied to the yaw-rate channel.

Class	Profile on ω_z_ (rad s^−1^)	Window (s)	Rationale
A1	0.4 sin(2π · 0.4 · (t − 20))	20–26	Oscillatory, maneuver-correlated; probes the attenuation gate
A2	+0.6 constant	55–65	Persistent bias; the classical false-data-injection case
A3	0.08 · (t − 72) ramp	72–80	Slow drift; probes the detectability floor

**Table 3:** Nomenclature and symbols used in Section 4, with definitions, units, and identified values, where applicable.

Symbol	Definition	Unit/Value
L	Wheelbase of the ego vehicle	3.005 m
K_us_	Understeer gradient, friction-conditioned	s^2^ m^−1^, Section 5.2
τ_s_	Steering-actuator lag time constant	s, Section 5.2
delta	Front road-wheel angle from the actuator command	rad
v	Longitudinal speed (operating variable)	m s^−1^
ω_z_	Measured yaw rate	rad s^−1^
r	Residual, measured minus predicted yaw rate	rad s^−1^
b, B	Operating-variable bin index and bin count	B = 18
n_min_	Minimum samples for a bin-local estimate	40
μ_r_, σ_r_	Bin-conditional residual mean and standard deviation	rad s^−1^
z	Residual standardized by OCRN	Dimensionless
τ_m_	Leaky-integrator membrane time constant	0.20 s
Vth	Encoder firing threshold	0.30
W	Causal window length	10 samples (100 ms)
p	Calibration percentile for the alarm threshold	99.0
n	Persistence requirement on consecutive exceedances	1
β_1_, β_2_	Attenuation-gate coefficients	0, 0 (selected)
w_a_	Lateral-acceleration channel weight	0 (selected)
k	Detectability-floor multiplier	0, 1, 2, 3
δ_min_	Minimum detectable fault, k σ_r_	rad s^−1^

**Table 4:** Nominal yaw-rate residual statistics by speed band (model M4, friction-conditioned).

Speed Band (m s^−1^)	n	σ_r_ (rad s^−1^)	Mean |r| (rad s^−1^)
0.0–0.5	104,900	0.0140	0.0019
0.5–2.0	5640	0.2455	0.1035
2.0–5.0	13,609	0.4121	0.1469
5.0–10.0	92,265	0.1214	0.0487
10.0–15.0	2236	0.0079	0.0028
Above 15.0	5988	0.0026	0.0008

**Table 5:** Scenario matrix. Conditions are labeled by their physical friction coefficient; the simulator weather preset is given for reproducibility.

μ	Weather Preset	Towns	Scenarios	Samples
1.00	Clear noon	Town01–Town05	5	≈45,000
1.00	Cloudy sunset	Town01–Town05	5	≈45,000
0.85	Custom dense fog (density 85, wetness 30)	Town01–Town05	5	≈45,000
0.65	Hard rain noon	Town01–Town05	5	≈45,000
0.35	Wet sunset	Town01–Town05	5	≈45,000

**Table 6:** Forward-model structure selection on attack-free data. Coefficients of determination are computed on samples above 2 m s^−1^, where both the predicted and measured yaw rates are non-degenerate; the leave-one-town-out figure for the learned correction is computed on the full corpus. Figure 2b is computed on all samples and its band statistics are those presented in Table 4.

Model	Fitted Parameters	RMSE (rad s^−1^)	R^2^
M1 kinematic bicycle, (v/L) tan δ	—	0.5481	−1.5303
M2 kinematic bicycle, small-angle	—	0.3830	−0.2356
M3 linear bicycle + understeer gradient	K_us_ = 0.0650	0.2512	0.4683
M4 = M3 + steering-actuator lag	K_us_ = 0.0350, τ_s_ = 0.50 s	0.2127	0.6188
M4, friction-conditioned parameters	see text	—	0.7107
M4 + learned correction (leave-one-town-out)	Ridge, 36 features	—	0.7374

**Table 7:** In-sample against leave-one-town-out identification of the friction-conditioned parameters on samples above 2 m s^−1^.

μ	n	K_us_ In-Sample	K_us_ LOTO (SD)	τ_s_ In-Sample	τ_s_ LOTO	R^2^ In-Sample	R^2^ LOTO	Optimism
0.35	19,370	0.030	0.034 (0.011)	1.00	0.94	0.4708	0.4246	+0.0462
0.65	25,644	0.055	0.056 (0.010)	0.30	0.28	0.6202	0.6082	+0.0120
0.85	23,731	0.030	0.030 (0.003)	0.40	0.40	0.8120	0.8042	+0.0078
1.00	45,353	0.015	0.015 (0.000)	0.10	0.09	0.9915	0.9910	+0.0005

**Table 8:** Detection performance, pooled across 25 scenarios and reported per scenario.

Condition	Coverage	Precision	Recall	F_1_	FAR Mean	FAR Worst	Worst F_1_
Unrestricted (k = 0)	100.0%	97.34%	76.35%	85.57%	0.76%	1.55%	21.90%
Floor-restricted (k = 3)	80.1%	97.30%	93.99%	95.62%	0.76%	1.55%	79.63%

**Table 9:** Detectability-floor sweep at the identified operating point.

k	Coverage	Precision	Recall	F_1_	Mean FAR	Worst F_1_
0	100.0%	97.34%	76.35%	85.57%	0.76%	21.90%
1	93.5%	97.34%	81.55%	88.75%	0.76%	26.79%
2	87.8%	97.33%	86.59%	91.65%	0.76%	36.52%
3	80.1%	97.30%	93.99%	95.62%	0.76%	79.63%

**Table 10:** Independent validation of the detectability floor. Recall within bands of the ratio of injection magnitude to the local residual standard deviation, computed without reference to the floor.

|a|/σ_r_	Injected Samples	Recall (%)
Below 1	3905	1.64
1 to 2	3385	3.10
2 to 3	4662	10.40
3 to 5	4897	50.54
5 to 10	5641	94.34
Above 10	37,475	99.61

**Table 11:** Channel-wise separability, measured as area under the receiver operating characteristic of the standardized residual magnitude.

Channel	Mean AUC	Worst-Scenario AUC
Yaw rate ω_z_	0.9647	0.8850
Lateral acceleration a_y_	0.8497	0.6620
Both channels, unweighted sum	0.9516	0.8630

**Table 12:** Recall and mean time-to-detect by attack class.

Class	Description	Recall (k = 0)	Recall (k = 3)	Coverage	Mean TTD
A1	Maneuver-correlated sinusoid	83.19%	96.4%	71.8%	78 ms
A2	Constant bias	99.50%	99.50%	100.0%	42 ms
A3	Slow ramp	80.96%	95.1%	66.3%	189 ms

**Table 13:** Effect of the threshold-calibration protocol; all other components are held as fixed.

Calibration Regime	Deployable	F_1_	Precision	Mean FAR
Startup window (first 18 s of deployment run)	Yes	93.18%	90.07%	3.10%
Offline nominal run, same surface condition	Yes	95.62%	97.30%	0.76%

**Table 14:** Component ablation at the identified operating point (k = 3). Each row modifies a single component of the full method.

Configuration	F_1_	Precision	Recall	Mean FAR	Worst F_1_
Full method (learned + OCRN + LIF, no gate)	95.62%	97.30%	93.99%	0.76%	79.63%
Minus learned correction (analytic model only)	95.53%	97.10%	94.00%	0.85%	65.24%
Minus OCRN (global σ)	73.84%	95.80%	60.08%	0.77%	0.00%
Minus LIF encoder (raw standardized magnitude)	95.44%	94.88%	96.01%	1.51%	82.14%
Plus attenuation gate (β_1_ = 1.8, β_2_ = 0)	95.39%	96.44%	94.36%	1.02%	76.23%
Plus attenuation gate (β_1_ = 1.8, β_2_ = 0.8)	95.76%	95.26%	96.26%	1.40%	78.63%
Minus friction-conditioned parameters	94.28%	97.33%	91.42%	0.68%	65.24%
Minus offline calibration (startup window)	93.18%	90.07%	96.51%	3.10%	75.03%

**Table 15:** Detection rule crossed with normalization at k = 3, under a mean false-alarm constraint of 2%. Each cell is the best configuration found by exhaustive search over the method’s design space.

Detector	Normalization	F_1_ Mean (%)	F_1_ Worst (%)	FAR Mean (%)	Feasible	Grid
Windowed chi-squared	Global σ	76.18	0.00	1.90	Yes	540
Windowed chi-squared	OCRN	94.74	81.75	1.14	Yes	540
CUSUM	Global σ	45.35	5.18	48.64	No	432
CUSUM	OCRN	48.60	11.30	59.37	No	432
Proposed (LIF encoder)	Global σ	79.86	12.23	1.99	Yes	10,800
Proposed (LIF encoder)	OCRN	94.83	73.33	1.55	Yes	10,800

**Table 16:** False-alarm rate, event rate, recall, and mean time-to-detect as a function of the persistence requirement; all other parameters held at the identified operating point.

Persistence n	FAR (%)	Events/Hour	Recall (%)	TTD A1 (ms)	TTD A2 (ms)	TTD A3 (ms)
1	0.760	309	93.99	78	42	189
3	0.644	175	93.25	101	62	224
10	0.469	152	91.22	171	132	288
20	0.328	120	88.43	266	330	395
40	0.230	104	83.08	487	602	530
100	0.144	77	69.63	1337	1135	1361

**Table 17:** The five weakest scenarios at k = 3, ordered by F_1_.

Scenario	μ	Coverage	Precision	Recall	F_1_
Town03, wet-sunset surface	0.35	37.64%	88.57%	72.33%	79.63%
Town04, hard-rain surface	0.65	52.61%	95.75%	69.82%	80.75%
Town04, wet-sunset surface	0.35	75.58%	97.52%	78.36%	86.90%
Town03, dense-fog surface	0.85	49.75%	94.10%	85.43%	89.55%
Town05, wet-sunset surface	0.35	88.25%	98.16%	83.05%	89.97%

**Table 18:** Per-scenario diagnosis of the coverage variation. Median residual scales within the injection windows and the occupancy of each speed band during those windows, for the five scenarios at μ = 0.85 and the group summary at each friction level.

Scenario/Group	μ	Coverage (%)	Median σ_r_	2–5 m s^−1^ (%)	5–10 m s^−1^ (%)
Town01 Dense Fog	0.85	70.63	0.043	2.33	44.46
Town02 Dense Fog	0.85	86.21	0.051	0.00	0.00
Town03 Dense Fog	0.85	49.75	0.162	1.75	56.58
Town04 Dense Fog	0.85	82.25	0.002	0.00	25.00
Town05 Dense Fog	0.85	12.67	0.234	10.13	74.58
Group Mean	1.00	93.87	0.002	2.44	-
Group Mean	0.85	60.30	0.051	2.84	-
Group Mean	0.65	77.67	0.067	6.31	-
Group Mean	0.35	74.59	0.025	11.04	-

**Table 19:** Results by held-out towns under the leave-one-town-out protocol, at k = 3.

Held-Out Town	Precision (%)	Recall (%)	F_1_ (%)	FAR (%)	Coverage (%)
Town01	98.34	96.39	97.35	0.48	80.80
Town02	96.96	96.71	96.84	1.01	91.32
Town03	96.02	93.95	94.98	1.00	70.44
Town04	97.34	89.13	93.06	0.72	80.83
Town05	97.78	93.37	95.52	0.59	76.90

**Table 20:** Nested leave-one-town-out selection. For each fold, the configuration is chosen on the other four towns and evaluated on the held-out town.

Held-Out Town	Inner-Fold F_1_ (%)	Held-Out F_1_ (%)	Precision (%)	Recall (%)	FAR (%)
Town01	95.70	97.78	98.77	96.81	0.36
Town02	95.99	96.52	94.64	98.47	1.86
Town03	96.16	95.87	95.25	96.51	1.24
Town04	96.49	89.34	95.22	84.14	1.24
Town05	96.27	95.47	92.76	98.34	2.15
Pooled (unbiased)	-	95.08	95.29	94.87	1.37
Single selection	-	95.62	97.30	93.99	0.76

## Data Availability

The telemetry corpus, CARLA scenario configurations, injection routines, monitor implementation, and scripts that reproduce the tables and figures in this paper are deposited in Zenodo under DOI 10.5281/zenodo.22341924 (https://doi.org/10.5281/zenodo.22341924). The code is released under the MIT license and the telemetry under CC BY 4.0.

## Abbreviation

The following abbreviations are used in this manuscript:

AUC	Area Under the Curve
CUSUM	Cumulative Sum
FAR	False-Alarm Rate
LIF	Leaky Integrate-and-Fire
LOTO	Leave-One-Town-Out
LPF	Low-Pass Filter
OCRN	Operating-Condition Residual Normalization
RMSE	Root Mean Square Error
SNN	Spiking Neural Network
TTD	Time-to-Detect
